# Understanding Race and Sex Differences in Age of Onset of Arteriolosclerosis with an MRI‐based in‐vivo Classifier

**DOI:** 10.1002/alz70856_101119

**Published:** 2025-12-25

**Authors:** Lianlian Du, Konstantinos Arfanakis, Arnold M Evia, Lisa L. Barnes, David A. A. Bennett, Alifiya Kapasi, Ana W. Capuano

**Affiliations:** ^1^ Rush Alzheimer's Disease Center, Rush University Medical Center, Chicago, IL, USA; ^2^ Wisconsin Alzheimer's Institute, University of Wisconsin‐Madison School of Medicine and Public Health, Madison, WI, USA; ^3^ Illinois Institute of Technology, Chicago, IL, USA

## Abstract

**Background:**

Brain arteriolosclerosis, a primary pathology of cerebral small vessel disease, is common in older adults and has been linked to lower cognitive and motor function, as well as an increased risk of dementia. Despite its prevalence, arteriolosclerosis can only be diagnosed at autopsy. Our previous work developed an MRI‐based in‐vivo classifier of arteriolosclerosis, ARTS. This study aims to estimate the age of onset of ARTS and improve our understanding of its longitudinal progression, particularly in individuals without dementia at baseline.

**Methods:**

Participants (*N* = 1,195) free of dementia at baseline were drawn from five longitudinal, community‐based clinical–pathological studies and imaged on two 3T MRI scanners. Gaussian Mixture Modeling (GMM) was applied to the last available ARTS score to identify groups with high and low ARTS scores. Demographics, baseline health, and cognitive performance were compared across groups. Longitudinal data were used to estimate onset age and disease duration based on ARTS scores. Sampled Iterative Local Approximation (SILA) modeled ARTS accumulation trajectories post‐onset of arteriolosclerosis. Onset differences were analyzed across scanners, sex, race, and pathology (Alzheimer's disease, atherosclerosis, and other neurodegenerative/vascular pathologies). We used linear mixed‐effects models to assess whether pathology, sex, and race modified ARTS trajectories.

**Results:**

The mean (SD) baseline MRI age was 82 (7.5) years, with 78.9% identifying as female. Among participants, 58.7% identified as non‐Latino White, 32.3% as non‐Latino Black, 8.1% as Latino, and 0.8% as other races/ethnicities. GMM identified an ARTS inflection point of 0.405, with 93.1% classified as low ARTS and 6.9% as high ARTS. The high ARTS group was older and had more women. The mean onset age was 93 (7.5) years. Females had a 6.6‐year earlier onset than males, while White participants had a 4‐year later onset than Black and Latino participants. Arteriolosclerosis, atherosclerosis, and macroscopic infarcts were associated with earlier onset. Males, Black participants, and those with severe arteriolosclerosis had faster ARTS accumulation. Sensitivity analysis confirmed similar results.

**Conclusion:**

Men and White participants had a later ARTS onset than women, Black, and Latino participants. Faster ARTS accumulation was observed in males, Black participants, and individuals with severe arteriolosclerosis, atherosclerosis, or macroscopic infarcts, likely due to mixed pathology effects.

